# Immediate Breast Reconstruction for Inflammatory Breast Carcinoma: A Scoping Review

**DOI:** 10.1002/jso.27959

**Published:** 2024-10-17

**Authors:** Nikhil Davé, John P. Hajj, Shahnur Ahmed, Luci Hulsman, Nikhi P. Singh, Carla S. Fisher, Mary E. Lester, Aladdin H. Hassanein

**Affiliations:** ^1^ Division of Plastic Surgery Indiana University School of Medicine Indianapolis Indiana USA; ^2^ Division of Breast Surgery Indiana University School of Medicine Indianapolis Indiana USA

**Keywords:** IBC, immediate breast reconstruction, inflammatory breast carcinoma, scoping review

## Abstract

Inflammatory breast carcinoma (IBC) is an aggressive form of breast cancer involving skin lymphatics. Breast reconstruction traditionally has been delayed in IBC. Immediate reconstruction has been described in select patients. Studies evaluating the reconstructive and oncologic safety of immediate breast reconstruction in this patient population are limited and retrospective. The purpose of this study is to assess the current body of literature on immediate breast reconstruction in IBC patients to identify knowledge gaps. A scoping review was conducted using PubMed, Scopus, Embase, and Cochrane databases. Original articles that evaluated patients diagnosed with IBC who underwent immediate breast reconstruction were included. The search yielded 821 articles, of which 9 articles containing 1429 IBC patients were included for analysis. Immediate implant‐based reconstruction occurred in 12.2% (174/1429) of patients. Immediate autologous reconstruction occurred in 19.0% (272/1429). Immediate reconstruction with both autologous and implant‐based techniques was 4.5% (64/1429). Reconstruction type was not reported for 63.0% (899/1429) of patients. Postoperative complications occurred in 1.8% (26/1429) of patients. Local cancer recurrence was 14.3% (3/21) at 18.9 months. The mortality rate was 32.4% (131/404) at 22 months. Performance of immediate breast reconstruction can be safely performed from a reconstructive standpoint in select patients.

## 1 Introduction

Breast cancer is the second most common cause of cancer‐related death in women [1, 2]. Inflammatory breast carcinoma (IBC) is a rare subtype that involves skin lymphatics and accounts for up to 5% of newly diagnosed breast cancer cases annually in the United States [3]. Treatment of IBC typically consists of neoadjuvant chemotherapy, mastectomy with axillary dissection, and adjuvant chemoradiation, which has increased 5‐year overall survival from 40% to 68% for stage III disease [4]. Wide resection of involved breast skin during mastectomy may limit reconstructive options [5]. Historically, delayed breast reconstruction following adjuvant radiation has been typically performed with caution given a 30% locoregional recurrence rate at 3 years [6]. Breast reconstruction using autologous tissue has been favorable over implant‐based reconstruction due to a better aesthetic outcome in IBC patients who require adjuvant radiation [7, 8]. While the current National Comprehensive Cancer Network (NCCN) guidelines include that delayed reconstruction is the current standard for postmastectomy IBC patients, immediate breast reconstruction has been given consideration as an alternative reconstructive strategy given improvements in cancer treatments for patients with IBC [9]. However, there is a paucity of literature assessing immediate breast reconstruction in this patient population [5, 10, 11]. The purpose of this scoping review is to evaluate current evidence of immediate breast reconstruction in IBC patients to identify existing knowledge gaps.

## 2 Methods

### 2.1 Research Questions

Research questions were developed in accordance with the Joanna Briggs Institute (JBI) methodology for scoping reviews [12]. Patients who were diagnosed with inflammatory breast cancer and received immediate breast reconstruction were included in this review. The concept of this review was reconstructive approach and safety of the procedure. Outcomes included postoperative complications (skin necrosis, surgical‐site infection, implant loss), cancer recurrence, and patient mortality. Safety was determined based on rate of postoperative complications, locoregional cancer recurrence, and death from breast cancer.

The following research questions were investigated:

1. What evidence is available in the current literature regarding the safety of immediate breast reconstruction for inflammatory breast cancer?
2. What is reported on the approach to immediate breast reconstruction for inflammatory breast cancer?

### 2.2 Identification of Relevant Studies

Our search strategy utilized a set of search terms relevant to our research question and included full‐text peer‐reviewed original articles. Search terms were broad and included “inflammatory breast cancer AND reconstruction” for all databases. PubMed, Embase, Scopus, and Cochrane databases were used. No search restrictions were placed on the year of publication.

### 2.3 Inclusion and Exclusion Criteria

To maintain consistency and internal validity, review articles, publications written in a non‐English language, abstracts without articles available in full text were excluded in the preliminary screening process. Articles were also excluded if they before 2014, were news articles, special topics, commentaries, letters, and editorials. Inclusion criteria included full‐text, peer‐reviewed, original articles that reported findings on patients diagnosed with IBC who underwent immediate reconstruction following mastectomy (Table 1).

**Table 1:** Full‐text selection criteria.

Subject	Inclusion	Exclusion
Article type	Any article containing information on IBC patients undergoing breast reconstruction	Any article that does not have information related to breast reconstruction in patients with IBC
Surgical procedure method	Available data on immediate breast reconstruction in IBC patients	Articles containing only delayed breast reconstruction and/or immediate chest wall reconstruction data

### 2.4 Data Extraction and Synthesis

**Figure 1:**
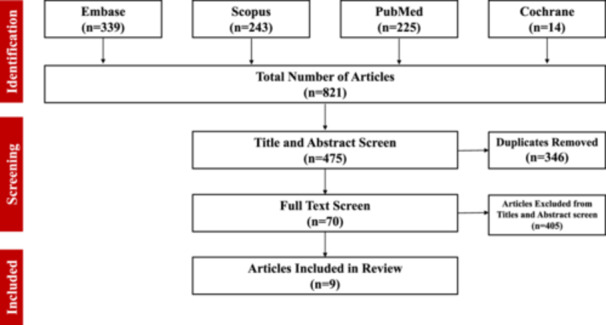
Preferred Reporting Items for Systematic reviews and Meta‐Analyses extension for Scoping Reviews (PRISMA‐ScR) Diagram Screening Process.

After completion of the literature search, relevant studies were imported into Covidence Systemic Review Software (Veritas Health Innovation, Melbourne, Australia) to enhance the screening and selection process. Title, abstract, and full‐text screening were independently completed by two reviewers and conflicts were resolved by a third author. The results of our search strategy, screening process, and full‐text selection were reported through a Preferred Reporting Items for Systematic Review and Meta‐Analyses (PRISMA) extension for a scoping review diagram (Figure 1) [13].

Data extraction of the chosen studies included the categories publication year, patient sample size, surgical techniques (mastectomy type, reconstruction method), and postoperative outcomes (complications, cancer recurrence, patient status). Relevant data not specifically mentioned in the article was marked as not reported.

## 3 Results

There were 821 articles found by the search terms used across PubMed, Embase, Scopus, and Cochrane databases. After screening and selection, nine articles met inclusion criteria [7, 10, 11, 14, 15, 16, 17, 18, 19]. Articles were excluded if they did not contain relevant data concerning surgical techniques or postoperative measures in IBC patients undergoing immediate breast reconstruction. These excluded articles focused on delayed breast reconstruction and immediate chest wall reconstruction in the selected group of patients with or without IBC. Articles that had delayed breast and immediate chest wall reconstruction were included only if distinct data on immediate breast reconstruction was available in IBC patients. A total of 1429 IBC patients were included in our review population from the nine studies (Table 2). Detailed evaluation of each included article was performed (Table 3). Information not present was marked as not specified. A tabulated summary of the extracted data from the nine selected articles was performed (Table 4). Treatment of IBC followed a standardized trimodal therapy consisting of neoadjuvant chemotherapy, modified radical mastectomy (MRM), radiation therapy in all studies. Immediate autologous breast reconstruction occurred in 19.0% (272/1429) of patients [7, 11, 16, 17, 19]. Immediate breast reconstruction using a tissue expander or implant occurred in 13.6% (194/1429) of patients [7, 11, 15, 16]. Reconstruction type was not specified in 63.0% (899/1429) of patients [7, 10, 14, 16, 18, 19]. Postoperative complications (skin necrosis, surgical‐site infection) were reported in 1.8% (26/1429) of patients [10, 11, 15, 16, 17]. Positive margins in IBC patients who underwent immediate breast reconstruction were discussed in 2 studies and were present in 9% (59/651) [7, 11]. Among studies that reported data on mortality, death from breast cancer occurred in 32.4% (131/404) with one reported study at median time of 21.9 months (range 1.8–10 years) [11, 14]. Among the studies that examined cancer recurrence, local recurrence in those who underwent immediate breast reconstruction was 14.3% (3/21) at 18.9 months postoperatively [11, 16, 17, 19]. The mean follow‐up of the studies was 4.28 ± 1.4 years.

**Table 2:** Review articles and included patient cohort size.

Author	Year	Institution	Patient population
Brown et al.	2014	United States	4
Simpson et al.	2016	United States	16
Patel et al.	2018	United States	44
Nakhlis et al.	2019	United States	13
Wang et al.	2019	China	310
Zhou et al.	2020	China	7
Smolanka et al.	2021	Ukraine	12
Hoffman et al.	2021	United States	635
Nair et al.	2022	Canada	388

**Table 3:** Extracted data from included review articles.

Author	Year	Type of mastectomy	Type of reconstruction (flap type/tissue expander)	Complications	Cancer recurrence	Outcomes
Brown	2014	4 ‐> Modified radical mastectomy	3 ‐> Bilateral reconstruction with tissue expander placement at the time of mastectomy 1 ‐> Placement of a contralateral tissue expander only	1 ‐> After delayed permanent implant placement, the patient developed extrusion of the implant 3 years after radiation therapy.	Not specified	Not specified
Simpson	2016	6 ‐> contralateral prophylactic mastectomy (CPM) 10 ‐> Unilateral mastectomy (MRM)	13 ‐> Tissue expanders placed 3 ‐ > Autologous	2 ‐> Infection requiring intravenous antibiotics 1 ‐> Expander removal 2 ‐> Tissue loss	7 ‐> Metastatic recurrence	7 ‐> Dead of disease
Patel	2018	44 ‐> Not specified	44 ‐> Not specified	3 ‐> Mechanical complications with the implant	Not associated with elevated recurrance	Reconstruction Status did not effect mortality
Nakhlis	2019	13 ‐> Modified radical mastectomy	3 ‐> Tissue expander 3 ‐> Single‐stage implants 1 ‐> Deep inferior epigastric perforator flap (DIEP) 4 ‐> Transverse rectus abdominis mycutaneous flap (TRAM) 1 ‐> Latissimus dorsi flap 1 ‐> Unspecified	1 ‐> Transverse rectus abdominis mycutaneous flap had flap necrosis, reconstruction was ultimately salvaged 5 ‐> Reoperations due to minor esthetic issues like fat necrosis and capsular contracture	2 ‐> Locoregional recurrence alone 9 ‐> Distant disease recurrence 1 ‐> Locoregional recurrence and distant matastasis concurrently	Median disease free survival was 14 months (range 1–63 months)
Wang	2019	310 ‐> Not specified	310 ‐> Not specified	Not specified	Not specified	IBR exhibited similar survival outcome whether the patients had IBC or not
Zhou	2020	6 ‐> Modified radical mastectomy 1 ‐> Lumpectomy	1 ‐> Patient with lumpectomy underwent latissimus dorsi flap 6 ‐> Not specified	Not specified	1 ‐> Had distant metastases before surgery	Not specified
Smolanka	2021	12 ‐> Radical mastectomy	11 ‐> Transverse rectus abdominus myocutaneous flap (TRAM) 1 ‐> Muscle sparing—transverse rectus abdominus myocutaneous flap (MS‐TRAM)	2 ‐> Marginal necrosis 1 ‐> Prolonged lymphorrhea 3 ‐> Corrective plastic interventions (removal of operative scars and contralateral mastopexy) 5 ‐> Nipple‐areola complex reconstruction	1 ‐ > Distant metastases in spine and small pelvis bones 1 ‐ > Regional recurrence in the displaced flap near postoperative scar	Not specified
Hoffman	2021	353 ‐> Unilateral mastectomy (MRM) 282 ‐> Bilateral mastectomy (MRM)	250 ‐> Autologous flap based technique 171 ‐> Implant based 64 ‐> Both tissue‐based and implant‐based 150 ‐> Not specified	Not specified	Not specified	Immediate breast reconstruction had better overall survival compared to no reconstruction
Nair	2022	217 ‐> Unilateral mastectomy (MRM) 171 ‐> Bilateral mastectomy (MRM)	388 ‐> Not specified	Not specified	Not specified	244 ‐> Alive 124 ‐> Death from breast cancer 8 ‐> Death from other cancer 3 ‐> Death from heart disease 6 ‐> Death to other diseases. 3 ‐> Unknown cause of death follow up time 6.0 years

**Table 4:** Number of reported patients with specific findings across studies.

*Type of mastectomy*	
Intervention	Patients
Unilateral mastectomy (MRM)	580
Bilateral mastectomy (MRM)	453
MRM (unspecified)	23
Radical mastectomy	12
Contralateral prophylactic mastectomy	6
Lumpectomy	1
*Type of reconstruction*	
Intervention	Patients
Autologous (not specified)	253
Implant	174
Autologous (not specified) and implant	64
Tissue expander	20
TRAM	16
Latissimus dorsi	2
DIEP	1
*Complications*	
Complication	Patients
Corrective reoperation	13
Mechanical implant complications	4
Necrosis	3
Infection	2
Tissue loss	2
Expander removal	1
Lymphorrhea	1
*Cancer recurrence*	
Location	Patients
Metastatic	17
Local	3
Local and metastatic	1
*Outcomes*	
Status	Patients
Alive	244
Death from disease	131
Death from other cancer	8
Death from other disease (excluding heart)	6
Death from heart disease	3
Unknown cause of death	3

## 4 Discussion

IBC is an aggressive subtype of breast cancer that accounts for 1%–5% of new breast cancer cases in the United States [3]. A delayed approach to breast reconstruction using autologous tissue or implants after tissue expansion has been traditionally performed in IBC patients due to diffuse breast skin involvement requiring wide skin resection [20]. While immediate breast reconstruction performed in breast cancer patients can decrease psychosocial morbidity and improve quality of life, the role of immediate breast reconstruction has not been well‐elucidated in IBC patients [21]. While national guidelines recommend the performance of delayed breast reconstruction compared to immediate reconstruction in cases of IBC, rates of immediate reconstruction have risen from 6.2% in 2004 to 15.3% in 2015 in this patient population [14].

In this systemic scoping review, we found that among the nine included original studies, incomplete data was frequent. IBC patients were often a subgroup of patients and reporting of reconstruction was grouped with the other patients. This is partially due to the lack of distinction in current procedural (CPT) code for immediate versus delayed autologous reconstruction in major databases for patients with IBC. In addition, many authors only provided data on two or three of the areas of evidence shown in Table 3 and did not report reconstructive approach or postoperative complications. In a study by Wang et al., 310 IBC patients who underwent immediate breast reconstruction were included, but this study only reported that there was no difference in the survival of immediate breast reconstruction patients over a 4.4‐year follow‐up period between patients with or without IBC [18]. Given the paucity of literature, major gaps include the lack of specific reporting on the timing of reconstruction, the type of flap used, satisfaction with the cosmetic outcome, and specifics of patient survival.

Initially, the standard of care for IBC was surgical resection and was associated with poor survival outcomes [4]. These early findings illustrated IBC as being aggressive with a poor prognosis with an average survival time of 22 months [14, 22]. A multimodal therapeutic approach consisting of neoadjuvant and adjuvant treatment, in addition to mastectomy and axillary dissection, has significantly improved the survivability of this condition [3]. Locoregional recurrence was assessed to be 25.5% in this review of IBC patients who underwent immediate breast reconstruction. A retrospective study of 23 IBC patients compared immediate versus delayed reconstruction and found a 29% locoregional recurrence rate in the immediate group versus 33% locoregional recurrence in the delayed reconstruction group [23]. In this scoping review, we report a mortality rate of 32.4% from inflammatory breast cancer who underwent immediate breast reconstruction. A study performed by Nair et al. observed a 10‐year survival rate of 62.2% of IBC patients who did not receive reconstruction compared to 56.6% of IBC patients who underwent breast reconstruction [14]. Previously, breast reconstruction was thought to add unnecessary exposure of risk for these patients who would not gain from the long‐term benefits of the procedure. However, this concept is evolving as evident by 1429 patients who received immediate breast reconstruction in this review. Furthermore, this number only represents a portion of the total reconstruction performed in IBC patients. The quality‐of‐life benefit of breast reconstruction has been well documented with statistically higher scores in social functioning and mental health reported on a 36‐item short‐form survey administered to patients immediately postoperatively and at 1‐year follow‐up [14, 24]. The improved longevity of IBC patients is exciting and opens new opportunities to improve the psychosocial burden of these patients over time, including breast reconstruction.

Despite evidence supporting the feasibility and efficacy of immediate reconstruction in IBC, delaying reconstruction currently still remains most common [2]. A 2018 study conducted an international survey of IBC physician experts at high‐volume centers record their thoughts on immediate versus delayed reconstruction in IBC patients [4]. The study found that 82% answered that they did not believe it appropriate to perform immediate breast reconstruction in IBC and that delayed breast reconstruction was the appropriate approach [4]. The primary reasons given for this conclusion revolved around postmastectomy radiotherapy [4]. Concerns included the endangered deep chest wall and intermammary lymph nodes not receiving adequate radiotherapy due to the superficial reconstructed tissue, and poor cosmetic outcomes given the possible deterioration of the reconstruction upon exposure to radiotherapy [4]. The prevalence of cautious philosophy toward reconstruction in this population is further evidenced by only 27% of participants endorsing the use of tissue expanders at initial mastectomy to allow for future breast reconstruction [4]. Delayed reconstruction following radiation without preserving the breast skin requires autologous reconstruction to reconstruct the deficient skin. However, staged, delayed autologous reconstruction with immediate tissue expander placement has become more common [25, 26]. Preserving and expanding breast skin when the skin involved has been removed may preserve an implant‐only reconstruction which otherwise would not be feasible.

## 5 Conclusion

Though delayed breast reconstruction in IBC patients remains the current paradigm, immediate breast reconstruction is being performed and recent evidence is supporting its feasibility and safety. Immediate breast reconstruction has many benefits including improving patient psychosocial outcomes and quality of life. The effectiveness of trimodal therapy in providing IBC patients with longevity necessitates a second look at immediate breast reconstruction. This scoping review identifies that there is a paucity in the literature regarding the findings of patients undergoing breast reconstruction for IBC. This scoping review demonstrates that the optimal timing remains as a knowledge gap for breast reconstruction in IBC patients. There may be a subset of patients in which immediate reconstruction with a tissue expander may be advantageous if enough skin remains to close.

## Conflicts of Interest

The authors declare no conflicts of interest.

## Synopsis

Inflammatory breast carcinoma (IBC) is an aggressive form of breast cancer involving skin lymphatics and breast reconstruction traditionally has been delayed in IBC patients. This scoping review identifies that there is a paucity in the literature regarding the findings of IBC patients undergoing breast reconstruction and demonstrates that the optimal timing remains as a knowledge gap for breast reconstruction in IBC patients.

## Data Availability

The data that supports the findings of this study are available from the corresponding author upon reasonable request.
